# Automatic extraction of facial median sagittal plane for patients with asymmetry based on the EDMA alignment algorithm

**DOI:** 10.1186/s13005-024-00430-4

**Published:** 2024-05-18

**Authors:** Yujia Zhu, Aonan Wen, Ning Xiao, Zixiang Gao, Shengwen Zheng, Xiangling Fu, Yijiao Zhao, Yong Wang

**Affiliations:** 1 Center of Digital Dentistry, Department of Prosthodontics, Peking University School and Hospital of Stomatology, Beijing, China; 2 National Center of Stomatology, Chengdu, China; 3 National Clinical Research Center for Oral Diseases, Chengdu, China; 4 National Engineering Research Center of Oral Biomaterials and Digital Medical Device, Beijing, China; 5 Beijing Key Laboratory of Digital Stomatology, Beijing, China; 6 NHC Research Center of Engineering and Technology for Computerized Dentistry, Beijing, China; 7 School of Computer Science, Beijing University of Posts and Telecommunications (National Pilot Software Engineering School), Beijing, China; 8 Key Laboratory of Trustworthy Distributed Computing and Service, Ministry of Education, Beijing University of Posts and Telecommunications, Beijing, China

**Keywords:** Facial asymmetry, Median sagittal plane, Procrustes analysis, Euclidean distance matrix analysis, Weights

## Abstract

**Background:**

We aimed to establish a novel method for automatically constructing three-dimensional (3D) median sagittal plane (MSP) for mandibular deviation patients, which can increase the efficiency of aesthetic evaluating treatment progress. We developed a Euclidean weighted Procrustes analysis (EWPA) algorithm for extracting 3D facial MSP based on the Euclidean distance matrix analysis, automatically assigning weight to facial anatomical landmarks.

**Methods:**

Forty patients with mandibular deviation were recruited, and the Procrustes analysis (PA) algorithm based on the original mirror alignment and EWPA algorithm developed in this study were used to construct the MSP of each facial model of the patient as experimental groups 1 and 2, respectively. The expert-defined regional iterative closest point algorithm was used to construct the MSP as the reference group. The angle errors of the two experimental groups were compared to those of the reference group to evaluate their clinical suitability.

**Results:**

The angle errors of the MSP constructed by the two EWPA and PA algorithms for the 40 patients were 1.39 ± 0.85°, 1.39 ± 0.78°, and 1.91 ± 0.80°, respectively. The two EWPA algorithms performed best in patients with moderate facial asymmetry, and in patients with severe facial asymmetry, the angle error was below 2°, which was a significant improvement over the PA algorithm.

**Conclusions:**

The clinical application of the EWPA algorithm based on 3D facial morphological analysis for constructing a 3D facial MSP for patients with mandibular deviated facial asymmetry deformity showed a significant improvement over the conventional PA algorithm and achieved the effect of a dental clinical expert-level diagnostic strategy.

## Background

Symmetry and harmony are key elements of an attractive and aesthetically pleasing face, and because of the biological and environmental factors in development, few symmetrical faces exist [1–3]. The clinical manifestations of facial asymmetries are diverse and complex and are most often found in the lower third of the face [4]. With the development of digital diagnostic and therapeutic techniques, three-dimensional (3D) facial symmetry analysis has become a fundamental part of orthognathic surgery and orthodontic treatment planning and an important part of aesthetic prosthodontics [5–7]. The correct orthognathic median sagittal plane (MSP) significantly influences the treatment outcome. Constructing a 3D facial MSP is a prerequisite and key to 3D facial symmetry analysis.

Traditional methods of constructing the MSP are often based on important anatomical landmarks on a 3D digital face model, and the selection of anatomical landmarks on the face is the core of this method [8, 9]. Previous studies have shown that the selection of landmarks has been based on different criteria, thereby complicating the achievement of a common approach for all facial deformities. Therefore, the method of constructing the MSP based on the overlap between the original and the mirror model of the 3D facial model (referred to as the original mirror alignment method) has received increasing attention in recent years [10]. The principle of the original mirror alignment algorithm is to optimally match the geometry of the 3D face model (original model) and its mirror model by overlapping them. The MSP of the original 3D face model was determined by analyzing the symmetry plane of the overlapping model. The core of the original mirror alignment method is the optimal overlap algorithm between the original 3D face and mirror images. Existing research mainly uses the iterative closest point (ICP) and Procrustes analysis (PA) algorithms [11–13]. The ICP algorithm has been used for many years in this field. This algorithm does not rely on anatomical landmarks, and the software can automate the full data matching and overlap between the original and mirror 3D facial models; however, in the case of complex facial deformities, an expert needs to assist in the manual screening of the non-deformed areas of the face; this is called “regional ICP algorithm” [14, 15]. The PA algorithm is based on the important anatomical landmarks of the face. Based on the one-to-one correspondence between the anatomical landmarks of the same name on the original 3D facial and mirror models, this algorithm achieves the minimum average distance between the original and the mirror sets of landmarks to obtain the optimal overlap position between the original and mirror models. The PA algorithm’s focus on important anatomical landmarks of the face is more in line with clinical experience and practice in dentistry and has proven to be more reliable [14]; however, it also suffers from poor suitability for complex facial deformities [16]. This is because the PA algorithm is not well suited to the needs of the patient. In 2021, Zhu et al. proposed a weighted Procrustes analysis algorithm to assign weights to landmarks, which was used to assess the clinical suitability of certain facial asymmetries [17]. This algorithm is clinically suitable in patients with facial asymmetry. However, the algorithm relies on commercial software for initial alignment, and the preprocessing process is complicated; this is not conducive to developing independent intellectual property rights of the algorithm program.

Based on previous research on weighted Procrustes analysis algorithms, this study uses the Euclidean distance matrix analysis (EDMA) method in morphological analysis to quantitatively evaluate and assign weights to anatomical landmarks. The EDMA-weighted Procrustes analysis (EWPA) method was developed for the automatic construction of the 3D MSP of the face. This study initially investigated the clinical suitability of the algorithm for cases of facial asymmetries, such as mandibular deviations, which are common in dental practice.

## Materials and methods

### Study participants

Forty patients with facial asymmetries of mandibular deviations were selected from the Department of Orthodontics, Department of Maxillofacial Surgery, and Department of Prosthetics of the Peking University Stomatology Hospital. The inclusion criteria were as follows: deviation of the chin from the midline of the face perpendicular to the line of the bilateral pupils via the point of the nasal root greater than 3 mm when the patients were in the natural head position [18, 19]. This study was approved by the Ethics Committee of Peking University School and Hospital of Stomatology (PKUSSIRB-202,054,042). The volunteers were fully informed of the content and purpose of the experiment and provided informed consent.

### Experimental equipment and software

A 3D facial scanner FaceScan (3D-Shape Corp, Germany) with a scanning speed of 0.2–0.8 s, scanning accuracy of 0.1 mm, range of 270–320° (covering the left ear to right ear), raster scanning principle, and charge-coupled device with 5 million pixels, and approximately 10,000 data points was used for the experiments. The mean distance between facial point clouds was within the range of 0.6–2 mm. The 3D facial data processing software Geomagic Studio 2013 (3D System, Morrisville, NC, USA) was used, the EWPA algorithm program was developed in the Python programming language, and the objective function of the classical PA algorithm was optimized for weighting.$\text{F}{\prime }$ is obtained as shown in Eq. (1):

1 $${\rm{F'}} = \mathop {min}\limits_Q \sum\nolimits_{i = 1}^p {{{\rm{W}}_{\rm{i}}}{{\left\| {{\rm{LMK}}\_{\rm{Or}}{{\rm{g}}_{\rm{i}}} - {\rm{Q}} \cdot {\rm{LMK}}\_{\rm{Mi}}{{\rm{r}}_{\rm{i}}}} \right\|}_2}},$$

where LMK_Org is the original landmark, LMK_Mir is the mirror landmark, LMK_Org_i_ and LMK_Mir_i_(i = 1,2,…,32) are the corresponding landmarks in the original and mirror point landmarks, respectively, Q is the spatial change matrix, p is the number of landmarks, and W_i_(i = 1,2,…,32) is the weight factor of each facial landmark.

### Experimental methods

#### Acquisition and processing of 3D face data

**Fig. 1:**
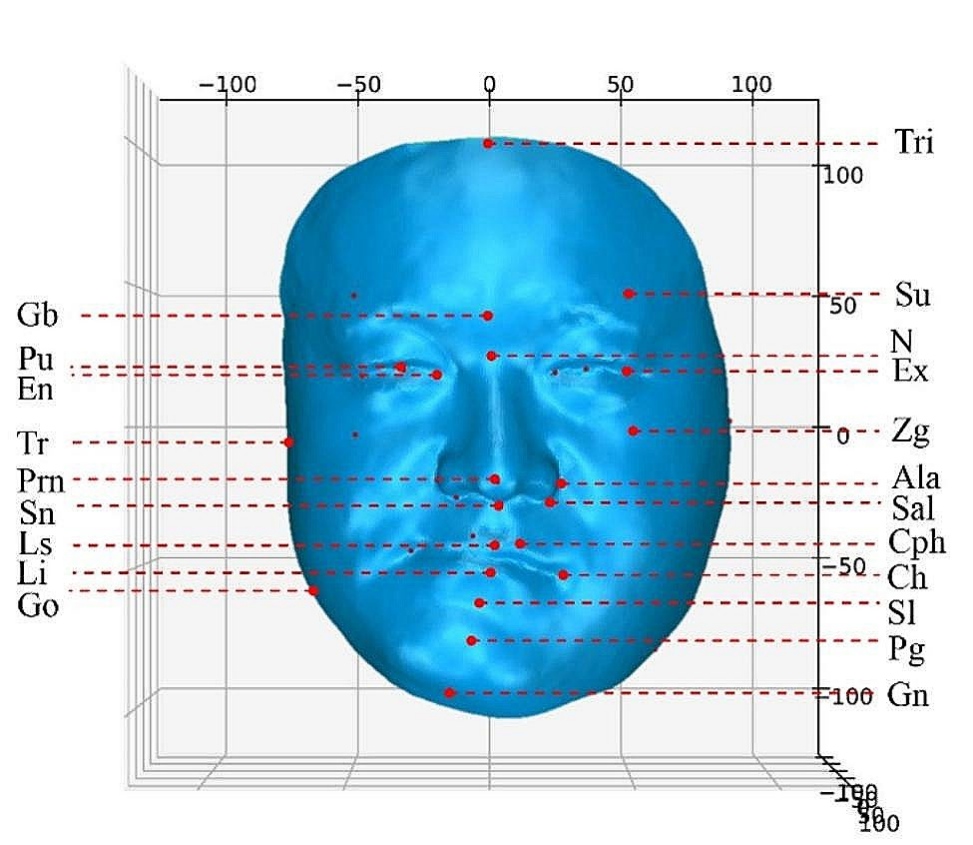
The 32 anatomic landmarks (upper facial third: trichion, glabella, superciliary ridge; middle facial third: nasion, pronasale, subnasale, exocanthion, pupil, endocanthion, tragion, zygion, alare, and subalare; lower facial third: labiale superius, labiale inferius, sublabiale, pogonion, gnathion, crista philtre, cheilion, and gonion)

The equipment was calibrated before acquisition to ensure that accurate data were obtained. The patient was seated 135 cm from the facial scanner and guided by the clinician to a natural head position, with the eyes looking forward, keeping the Frankfort horizontal plane (FH plane) parallel to the ground plane, and with a naturally relaxed facial expression. The criteria that could be adopted for 3D facial data were effective in displaying the facial contours, high resolution, and absence of significant movement and occlusion. Using the reverse engineering software Geomagic Studio 2013, the 3D data of the patient’s face were processed as necessary, including removing redundant data for hole repair. The original 3D facial model was adjusted to the natural head position such that the FH plane of the natural head coordinate system coincided with the XZ plane of the global coordinate system, and the sagittal plane coincided with the YZ plane of the global coordinate system. The extraction of important anatomical landmarks of the original facial model (Model_Org) was conducted by a senior expert based on clinical experience. In total, 32 anatomical landmarks (10 in the midline and 11 bilateral) were manually selected by the expert in the full facial region, including the trichion, glabella, superciliary ridge, nasion, pronasale, subnasale, exocanthion, pupil, endocanthion, tragion, zygion, alare, subalare, labiale superius, labiale inferius, sublabiale, pogonion, gnathion, crista philtre, cheilion, and gonion, which were extracted three times at one-week intervals to obtain the mean value of the coordinates as LMK_Org, as shown in Fig. 1; Table 1. The coordinates of the center of gravity of LMK_Org were calculated, and the original model was translated until the center of gravity of LMK_Org coincided with the origin of the global coordinate system and was saved as an .OBJ file.

**Table 1:** Definitions of three-dimensional facial anatomical landmarks

No.	Landmark	Abbreviation
1	Trichion	Tri
2	Glabella	Gb
3	Nasion	N
4	Pronasale	Prn
5	Subnasale	Sn
6	Labialesuperius	Ls
7	Labialeinferius	Li
8	Sublabiale	Sl
9	Pogonion	Pg
10	Gnathion	Gn
11–12	Superciliare	Sci
13–14	Endocanthion	En
15–16	Exocanthion	Ex
17–18	Pupil	Pu
19–20	Zygion	Zg
21–22	Alare	Ala
23–24	Subalare	Sal
25–26	Tragion	Tr
27–28	Gonion	Go
29–30	Crista philtri	Cph
31–32	Chelion	Ch

#### Quantitative assessment of the asymmetry of facial landmarks

In this study, the asymmetry of 3D facial anatomical landmarks was quantitatively assessed using the EDMA method, which compares the geometric differences between two individuals based on a matrix of distances between a series of landmarks [20, 21]. For example, if K anatomical landmarks on an organism exist (1, 2, 3, 4 ……K), the distance lines d(1,1), d(1,2), … d(1,k), d(2,1), d(2,2), … d(2,k), … d(k,1), d(k,2), …d(k, k) between any two landmarks can be calculated for a total of k(k-1)/2 line segments. From this a form matrix (FM) of order k × k is formed, which can reflect the common characteristics of a particular data type and the individual differences between the same types. When analyzing the differences between two bodies of the same type, the ratio of the distance of the corresponding landmarks from the line segments in the FM of the two bodies can be formed into a new matrix called the form difference matrix (FDM) of the two bodies.

**Fig. 2:**
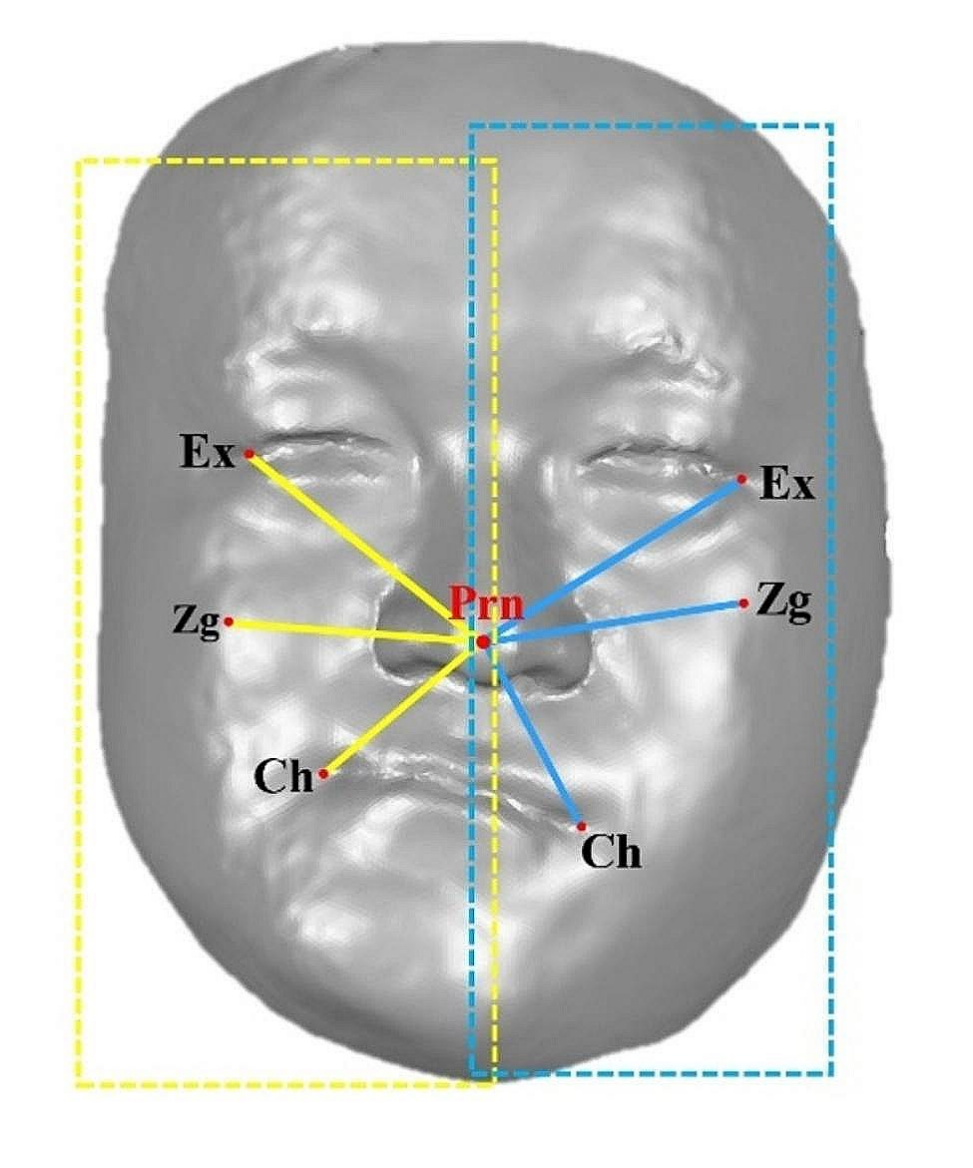
Line segment between landmarks (e.g., exocanthion, zygion, cheilion, pronasale). The yellow line segment is the distance between the midline and bilateral landmarks in the right side of the patient’s face landmark set, the blue line segment is the distance between the midline and bilateral landmarks in the left side of the patient’s face landmark set, and red points inside the blue and yellow dotted lines are the L landmark set and R landmark set respectively)

The specific needs of the quantitative evaluation of anatomical landmark asymmetry of the human face and the set of landmarks on the left and right sides of the face (11 landmarks each) were combined with the set of landmarks on the midline (10 landmarks) to build the “left face morphology” landmark set (L) and the “right face morphology” landmark set (R). “The “left face” and “right face” were treated as mirror images, and their morphological differences were quantified, and a new index based on the EDMA method was established to assess the degree of asymmetry of the landmarks, defined as morphological index E value. Considering landmark set R as an example, the right face morphological matrix was formed by calculating the distance between the midline and right landmark and each right landmark (a total of 10 × 1 + 11 × (11 − 1)/2 = 165 distances), named matrix ${M}_{R}$. Similarly, we calculated the left face morphology matrix${M}_{L}$ (a simplified diagram of the line segments in the matrix is shown in Fig. 2). The FDM was calculated as the ratio of the linear distances between the same name landmarks in the matrix ${ M}_{R}$ with ${M}_{L}$(the larger distance is used as the numerator; all ratios are greater than or equal to 1) forms of the left and right faces, with 165 ratio elements, as shown in Eq. (2):

2 $${\left(FDM\right)}_{k} :=\frac{max\left({M}_{Rk},{M}_{Lk}\right)}{min\left({M}_{Rk},{M}_{Lk}\right)},$$

where FDM is the morphological difference matrix of the left and right faces, and M_Rk_ and M_Lk_ are the kth elements of the matrices M_R_ and M_L_ (k = 1,2,…,165).

If the ratio in the matrix is 1, the left and right sides are perfectly symmetrical; if the ratio is greater than 1, a morphological difference (asymmetry) between the two sides is observed. To investigate the symmetry of a single landmark, this study proposes averaging a landmark and all its associated FDM line ratio elements as a morphological indicator of that landmark, defined as the F_i_ value (i = 1, 2, …,21), where F_i_ denotes the mean of the line segment ratio elements associated with the ith landmark in the morphological difference matrix. To make the distribution of the definition domain of F_i_ values as independent variables more consistent with the demand of the value domain of the assignment function, this study established a mapping relationship between the morphological index E values and Fi values, as shown in Eq. (3):

3 $${E}_{i}=100\left({F}_{i}-{F}_{min}\right),$$

where E_i_ is the morphological index of the landmark, F_i_ (i = 1,2,…,21) is the mean of the FDM line segment ratio elements of the landmark, and F_min_ is the minimum value of F_i_.

The smaller the E_i,_ the closer it is to 0, and the lower the degree of asymmetry of the landmark, and vice versa, the higher the degree of asymmetry. Two expressions of the assignment function were constructed in this study, namely, the offset power function and the linear function, as shown in Eqs. (4) and (5), respectively:

4 $$W = \frac{1}{{E + 1}},$$

5 $$W = - \frac{1}{{{E_{max}}}}E + 1.$$

**Fig. 3:**
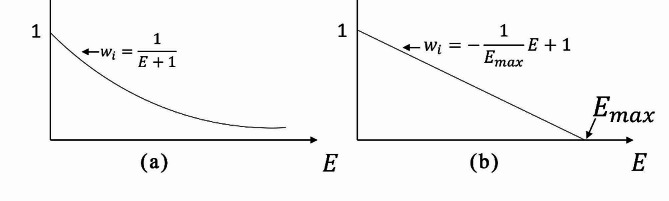
Function graphs of OP and LP. (**a**) Offset power function. (**b**) segmented power function. E is the morphological index of the landmark. W_i_ is the weight factor for each facial landmark

Graphs of the two assignment functions for the newly constructed landmark asymmetry index E are shown in Fig. 3.

In this study, W_i_ (i = 1,2,…,21) is the weighting factor of each facial landmark, E is the morphological index of the landmark, and E_max_ is the maximum value of E.

### Facial MSP construction

#### Experimental group 1: construction of the MSP based on the PA algorithm

For the 40 facial models in this study, the 3D spatial coordinates of the 32 landmarks in LMK_Org were input into the PA algorithm program based on the Python language based on the previous study of our research group [22]. LMK_Mir was obtained by mirroring the original landmark set based on the YZ plane and calculating the overlap effect between the original and mirror landmark set based on the PA algorithm in Python, without weight differences. The transformation matrix of the mirror landmark set was then calculated and loaded onto the LMK_Mir using Geomagic Studio 2013. Finally, the SRP of the facial data for each patient was constructed by taking the union of the original and mirror models (Model Uni_PA) in Geomagic Studio using the function “plane” and “symmetry”, defined as ‘MSP_PA’.

#### Experimental group 2: construction of the MSP based on the EWPA algorithm

The 3D spatial coordinates of the 32 landmarks in LMK_Org were input into the EWPA algorithm program based on the Python language, and the E value of each landmark was automatically calculated and weighted using the method described in Sect. 2.3.2. Based on the E value offset power function and linear function, W_i_ had a monotonically decreasing distribution with increasing E value; the highest weight was one, and the lowest was 0. The above algorithms for the weighting and alignment of landmarks based on the offset power function and linear function are defined as EOWPA and ELWPA, respectively.

The original landmark set is based on the YZ plane mirror landmark set, the weighted overlap effect of the paired landmarks between LMK_Org and LMK_Mir is achieved based on the weighted least squares method, and the final weighted optimal overlap position of LMK_Org and LMK_Mir is obtained. The same method in 2.4.1 was used to construct the MSP, defined as the EWPA MSP (MSP_EOWPA, MSP_ELWPA).

#### Reference group: construction of the reference plane based on the regional ICP algorithm

Based on the above Model_Org and Model_Mir models, a region of good symmetry of the face on the original model was manually selected by a senior expert on the Geomagic Studio software, a mirror model Model_Mir was obtained for Model_Org based on the mirrored YZ plane, and ICP registration was performed based on the region selected by the expert. After overlapping the regions of the original and mirror models, the original-mirror model was obtained, and the MSP was calculated, which was defined as the “reference plane” in this study (MSP_Ref). The final reference plane was constructed for the facial data of the 40 patients included in this study.

**Fig. 4:**
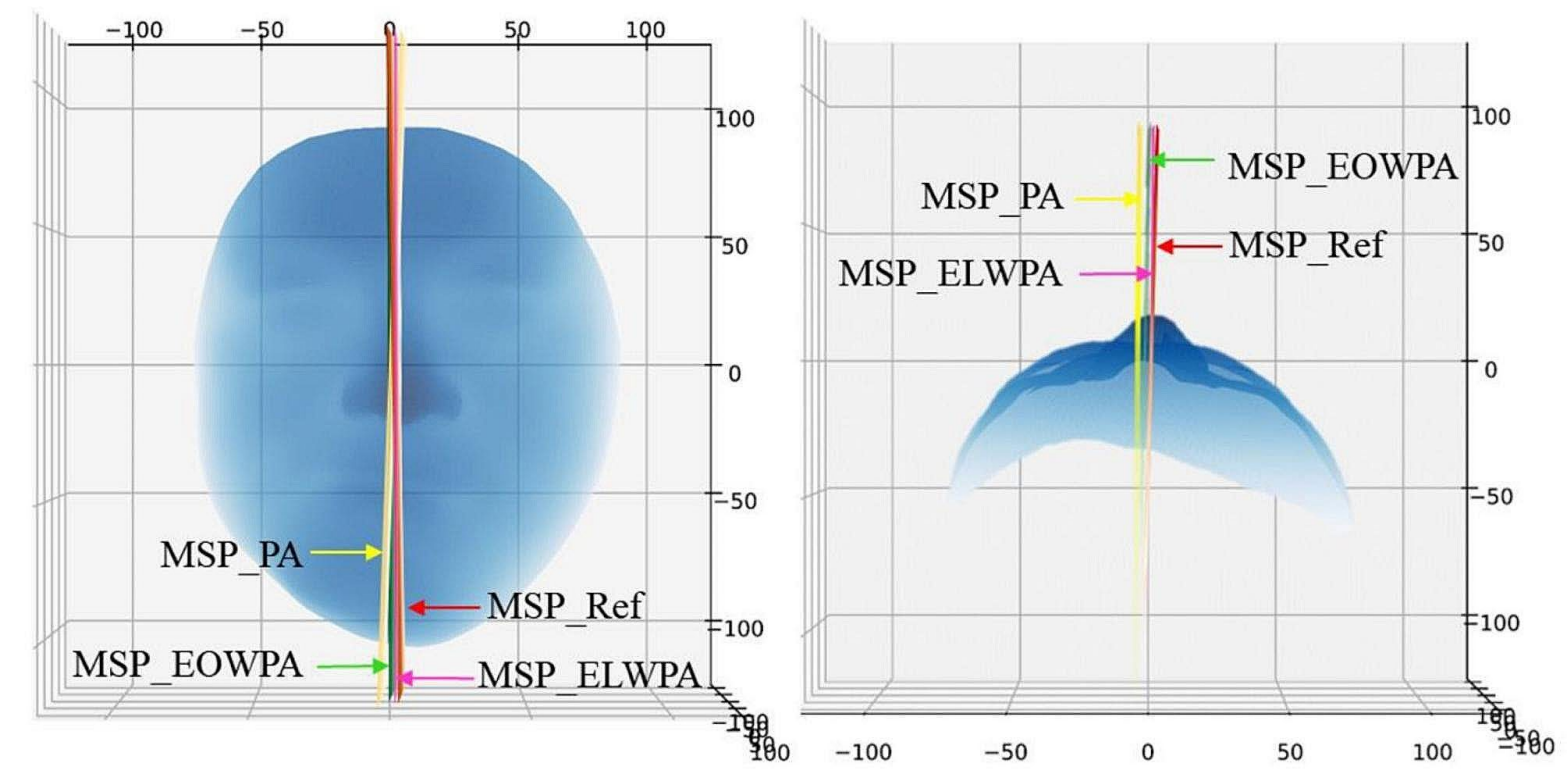
Determining the MSP based on the EOWPA, PA, and regional ICP algorithms in one case. The red plane signifies the MSP of ground truth, the green plane represents the EOWPA algorithm, and the yellow plane represents the PA algorithm

The effect of the same 3D face data based on the PA, EOWPA, ELWPA, and regional ICP algorithms on the construction of the MSP is shown in Fig. 4.

### Data analysis

To investigate the intra-observer error of landmark extraction, the senior clinical expert repeated the landmark measurements three times at one-week intervals, and the intra-class coefficient (ICC) was calculated. For the 40 facial models in this study, the angle between the MSP (MSP_PA, MSP_EOWPA, and MSP_ELWPA) and the expert reference plane (MSP_Ref), denoted as Ang_PA, Ang_EOWPA, and Ang_ELWP, respectively, constructed by the PA algorithm and the EWPA algorithm were calculated for each model. The mean and standard deviation of the angle error of each algorithm were calculated.

The Shapiro–Wilk normality test was performed using SPSS software (version 21.0) on the plane angle errors of experimental group 1 (PA algorithm) and experimental group 2 (EWPA algorithm) for the 40 patients. One-way ANOVA with Tukey’s multiple comparison test was used if the samples conformed to a normal distribution and the variances were homogeneous; otherwise, the Kruskal–Wallis H-test was used, and the test levels were all α = 0.05 on both sides as a significant difference.

The reference plane was used to classify the degree of facial asymmetric deformity in the 40 patients, based on the calculation of the distance from the pogonion to the reference plane and to analyze the angle error between the reference planes and MSP_PA, MSP_EOWPA, and MSP_ELWPA for different degrees of deformity in the 3D facial data.

## Results

**Fig. 5:**
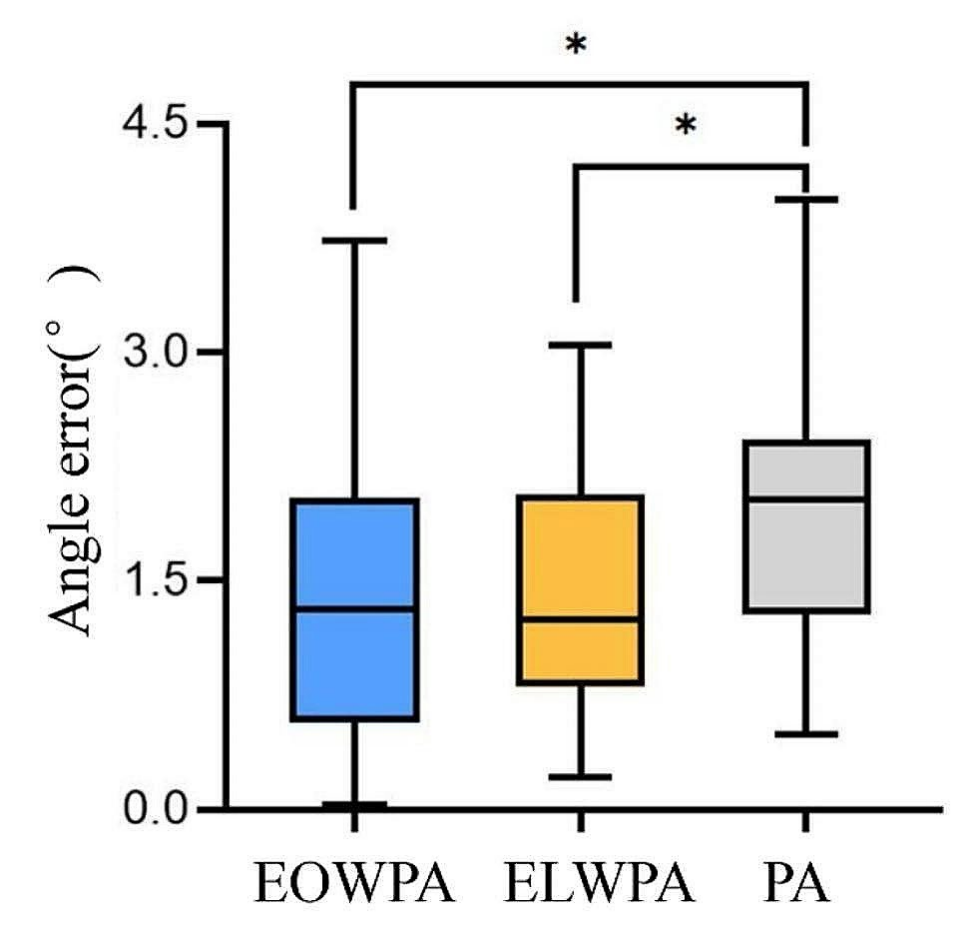
Boxplot of angle error for the EOWPA, ELWPA, and PA algorithms. The black asterisks signify *p* < 0.05

For all analysed measures of landmark detection by one expert, the intra-observer ICC values were>0.95 (0.97–0.99), demonstrating high intra-observer reproducibility. For the angle error (Ang_PA, Ang_EOWPA, and Ang_ELWP) in the MSP of the 40 facial models in this study in Experimental Group 1 (PA algorithm) and Experimental Group 2 (EWPA algorithm), the *p*-values of the Shapiro–Wilk normality test for all three groups were > 0.05, and they all conformed to a normal distribution. The EOWPA, ELWPA, and PA algorithm groups showed significant differences (*p* < 0.05), and the Tukey’s multiple comparison test showed significant differences in the angle errors between the PA algorithm group and the EOWPA and ELWPA algorithm groups. The mean and standard deviation of the angle errors of the EOWPA, ELWPA, and PA algorithm were 1.39 ± 0.85°, 1.39 ± 0.78°, and 1.91 ± 0.80°, respectively. The mean and standard deviation of the angle error for both EWPA algorithm groups were smaller, indicating that the MSP constructed by the EWPA algorithm was closer to the reference plane for the 40 cases of mandibular deviation in this study. The relevant sample data are presented in Fig. 5.

The 40 patients with mandibular deviation in this study were classified into three asymmetry degrees (AD): 14 patients with I degree (3 mm < AD ≤ 5 mm), 13 patients with II degree (5 mm < AD ≤ 8 mm), and 13 patients with III degree (AD>8 mm). The mean and standard deviation of the angle error of the PA, EOWPA, and ELWPA algorithms were obtained for each degree of patient, and the results of the measurement analysis are shown in Table 2. The two EWPA algorithms performed best in patients with moderate facial asymmetry, and in patients with severe facial asymmetry, the angle error was below 2°, which was a significant improvement over the PA algorithm.

**Table 2:** Angle error distribution based on EOWPA, ELWPA, and PA algorithm group for different degrees of patients with mandibular deviation

Mandibular deviation degrees	Number	Angle error (°)		
		EOWPA	ELWPA	PA
3–5 mm	14	1.28 ± 0.92	1.21 ± 0.77	1.70 ± 0.81
5–8 mm	13	1.14 ± 0.78	1.18 ± 0.66	1.65 ± 0.56
>8 mm	13	1.75 ± 0.77	1.78 ± 0.80	2.38 ± 0.83

## Discussion

### Feasibility of the EDMA method for quantitative assessment of asymmetry of facial landmarks

It has been indicated that the esthetics of the soft tissue plays a leading role in the selection of the therapeutic strategy; thus, the evaluation of facial asymmetry is of great significance for clinical treatment. Lee et al. found that mandibular deviation was significant factor affecting the assessment of facial asymmetry [7]. For patients with mandibular deviation, the novel EWPA algorithm provided a more suitable MSP for their 3D facial model, which achieved a result approaching that of the regional ICP algorithm. The EWPA algorithm established in this study is based on the EDMA in 3D morphometric analysis within the framework of the original mirror alignment algorithm. It quantitatively assesses the degree of asymmetry of anatomical landmarks. Reflecting the difference in their contribution to construct the MSP by assigning weights to the landmarks is an important innovation in this study. The EDMA method has been reported to be applied to craniofacial morphometry [23, 24], gender dimorphism studies [25, 26], and orthodontic arch morphometry analysis [27–29]. The principle of the EDMA method is to reflect the shape and size of an individual through a matrix of Euclidean distances between landmarks on the geometry of the individual and to reflect the morphological differences between individuals through the ratio of the corresponding matrix elements between individuals. Nie et al. [30] analyzed the morphology of the malocclusion arch and identified the landmarks that contributed relatively more to the morphological differences by removing them. In this study, we used the principles of inter-individual variation analysis using the EDMA method to construct the L and the R by combining the left and right landmark sets with the distance ratio. The L and R were treated as mirror models of each other, and the quantitative analysis of the difference between the left and right face morphology was carried out using the EDMA method to quantitatively analyze the asymmetry of the landmarks. The F_i_ value is obtained by calculating the average of all line ratio elements associated with the landmark, which directly reflects the contribution of the individual characteristics of the landmark to the morphological difference between the left and right faces. The higher the F_i_ value, the higher the degree of asymmetry of the landmark. Based on the analysis of the 3D facial data of 40 patients with mandibular deviation in this study, the algorithm assigned higher weights to upper-facial and mid-facial landmarks such as the nasion, pronasale, endocanthion, and exocanthion, while the algorithm assigned lower weights to the lower-facial landmarks such as the pogonion and gnathion gonion. The results of this algorithm are more in line with the clinical experience of dental clinics in diagnosing mandibular deviation.

### Weighted landmarks based on the EWPA algorithm are more in line with the diagnosis and treatment in dental clinics

In a review of previous studies on original-mirror alignment in the 3D facial median sagittal plane, the alignment algorithms were mainly based on ICP and PA algorithms [11, 13]. The ICP algorithm does not refer to the anatomical landmarks. Although certain scholars have demonstrated the reliability and reproducibility of the ICP algorithm in constructing the MSP for normal facial data, this method is less effective for poorly symmetric data [15]. Subsequently, the global ICP algorithm was improved by manually selecting facial regions with good symmetry regions for original and mirror overlap, which improved the clinical suitability of the ICP algorithm to an extent [31, 32]. The regional ICP algorithm introduces human intervention, which improves accuracy but also reduces the degree of automation of the algorithm. In this study, the regional ICP algorithm was used as the reference plane to evaluate the accuracy of the EWPA algorithm, considering that the algorithm currently clinically well-accepted [14, 31].

The PA algorithm considerably differs from the ICP algorithm in that it focuses more on the reference of facial anatomical landmarks. The core aim of the PA algorithm is to guide the overlap of the original mirror model based on anatomical landmarks to obtain the median sagittal plane. The principle of this algorithm is more in line with the clinical practice and experience of dentists and has received increased attention in recent years. The PA algorithm has been demonstrated to be well-suited for patients with no significant facial asymmetry [18]. However, complex facial deformities of poorly symmetrical PA landmarks, called the Pinocchio effect, are observed in the ICP algorithm [33]. One of the directions for improving the existing PA algorithm is screening PA landmarks. Gateno et al. [34] used the recursive PA algorithm to sort landmarks, remove obvious asymmetric landmarks, and use the remaining landmarks for PA operations, thus avoiding the interference of undesirable landmarks. Another potential improvement to the PA algorithm is the assignment of weights to different landmarks. Claes et al. [32, 35, 36] used a dense sequence of landmarks as a facial mask, mapped it onto the patient model, iteratively screened outliers, overlapped the original with the mirror mask using the PA algorithm, and assigned weights based on the matching quality of each landmark.

This study proposes an innovative EWPA algorithm based on 3D geometric morphological analysis to compile an automated algorithm to assess the symmetry of landmarks quantitatively, assign personalized feature weights to landmarks, and improve the construction of the MSP. In this study, by calculating the angle between the EWPA algorithm MSP and the reference plane, the average angle error was less than 2° for 40 patients with mandibular deviation, and that the MSP constructed using the EWPA algorithm was closer to the reference plane defined by clinical experts than that from the PA algorithm. Wu et al. [37] showed that determining when the angle error between the two planes is greater than 6. The angle error between the EWPA algorithm and expert plane is below 2, which indicates that the accuracy of the MSP constructed by the EWPA algorithm is almost equal to that of the expert reference plane. In this study, EWPA algorithm has good clinical suitability for constructing 3D MSP for patients with I degree and II degree, the average angle error was about 1.2°; however, the performance of patients with III degree needs to be further improved. No significant difference was observed between the effects of between the two EWPA algorithms, and it is more recommended for the diagnosis and asymmetry analysis of patients with I and II degree facial mandibular deviation. Compared with the expert group algorithm based on the ICP algorithm of commercial software platforms, the EWPA algorithm in this study exhibits more optimized secondary development, which is conducive to grassroots promotion of digital diagnosis and treatment. The EWPA algorithm automates the construction of 3D facial MSP, which can reduce the workload, shorten the time required for digital design, reduce dependence on expert experience, enhance clinical diagnostics, and improve therapeutic efficiency and effectiveness. This algorithm will be integrated into digital design software for oral and maxillofacial surgery and orthodontics to further evaluate its effectiveness in oral clinical applications.

## Conclusions

This study is based on the EWPA algorithm for the construction of the 3D facial MSP, which has the advantage that it does not reduce the degree of automation of the algorithm (without human intervention) and simulates as much as possible the expression of expert experience on the reference value of anatomical landmarks. The application of the EWPA algorithm in this study initially verified that the algorithm is suitable for patients with mandibular deviation. Further statistical and measurement analyses are needed to expand the sample types and further analyze the suitability for different facial deformities and degrees of facial deformities to guide clinical application.

## Data Availability

Not applicable.

## Abbreviations

AD: asymmetry degree
EDMA: Euclidean distance matrix analysis
ELWPA: algorithm for the weighting and alignment of landmarks based on the linear power function
EOWPA: algorithm for the weighting and alignment of landmarks based on the offset power function
EWPA: EDMA-weighted Procrustes analysis
FDM: form difference matrix
FH: Frankfort horizontal plane
FM: form matrix
ICP: iterative closest point
MSP: median sagittal plane
PA: Procrustes analysis
